# Serratus Anterior Plane Block for Pain Management After Video-Assisted Thoracoscopic Surgeries: A Narrative Review

**DOI:** 10.3390/medicina61061010

**Published:** 2025-05-28

**Authors:** Shahab Ahmadzadeh, Macie A. Serio, Angela Nguyen, Drew R. Dethloff, Camille Robichaux, Chizoba N. Mosieri, Sahar Shekoohi, Alan D. Kaye

**Affiliations:** 1 Department of Anesthesiology, Louisiana State University Health Sciences Center at Shreveport, Shreveport, LA 71103, USA; 2 School of Medicine, Louisiana State University Health Sciences Center at Shreveport, Shreveport, LA 71103, USA; mas002@lsuhs.edu (M.A.S.);; 3 Department of Pharmacology, Toxicology, and Neurosciences, Louisiana State University Health Sciences Center at Shreveport, Shreveport, LA 71103, USA

**Keywords:** regional anesthesia, postoperative pain, serratus anterior plane block (SAPB), video-assisted thoracoscopic surgery (VATS)

## Abstract

Video-assisted thoracoscopic surgery (VATS) is a minimally invasive diagnostic and therapeutic procedure utilized in various thoracic conditions. VATS has grown in popularity with ever-expanding knowledge of enhanced recovery after surgery (ERAS) protocols and its benefits regarding patient care and outcomes. Pain control following VATS is of utmost importance to minimize the complication risk. Options for pain control following VATS have traditionally included systemic IV analgesia but have evolved to include loco-regional analgesia as well. The serratus anterior plane block (SAPB) is one form of loco-regional analgesia utilized in VATS that has been shown to provide effective pain control of the anterolateral chest wall. Patients who received SAPB compared to control methods of anesthesia demonstrated significant decreases in postoperative pain and postoperative opioid consumption. SAPB is effective and offers a promising safety profile as the block is typically more superficial than other types of loco-regional analgesia. This review outlines the recent literature surrounding the use of SAPB for pain control in VATS.

## 1. Introduction

Video-assisted thoracoscopic surgery (VATS) is a type of minimally invasive surgical procedure for thoracic conditions that has evolved considerably in recent decades. VATS involves the insertion of a small video camera and other surgical instruments via three to four small incision ports in the patient’s chest, that allows for visualization of the chest cavity [1]. Through visualization and manipulation of the contents of the chest cavity, surgeons can use VATS for both therapeutic and diagnostic purposes in a variety of conditions. VATS is growing in popularity as the standard of care in thoracic procedures due to the minimally invasive nature and opportunity for enhanced recovery after surgery (ERAS) [2,3]. In general, evidence has shown that VATS has fewer respiratory complications, shorter length of hospital stays, and lower pain scores post-operatively when compared to patients who have undergone thoracotomy [4,5].

Effective pain management following surgical procedures is important to optimize recovery and to improve patient satisfaction [6]. The major component of acute pain following thoracic procedures is attributed to intercostal incision which spans multiple layers, including the skin, subcutaneous tissue, muscles, and parietal pleura. Although VATS operations have significantly smaller incisions than thoracotomies, identical tissue layers are traversed during the operation, and reported pain following VATS is often described as moderate to severe. Therefore, post-operative pain following VATS is a significant factor to consider in the care of patients [7,8]. Strategies for effective pain management adopt a multimodal approach tailored to patients. However, there is no current unified consensus to the best strategy to achieve effective pain management [9]. The traditional approach to pain management after VATS emphasizes optimal analgesia and minimized opioid use through systemic and loco-regional analgesia. There are many pharmacological options to reach this goal.

Systemic intravenous (IV) analgesia can be achieved by paracetamol (acetaminophen), non-steroidal anti-inflammatory drugs (NSAIDs), opioids, N-methyl-D-aspartate (NMDA) receptor antagonists, dexamethasone, lidocaine, and gabapentanoids (e.g., gabapentin and pregabalin). Standard IV regimens for patients undergoing VATS include paracetamol and NSAIDs, and, if needed, either a strong opioid (patient-controlled analgesia [PCA]) or a weak opioid (scheduled). Promising benefits have been seen with perioperative acetaminophen/ibuprofen administration in offering significantly decreased analgesic needs in patients whose status is post-VATS operation, as demonstrated by a double-blind randomized control trial by Lee et al. [8]. Loco-regional analgesia can offer longer pain relief and is of increasing interest in patients who have undergone VATS.

Historically, thoracic epidural analgesia (TEA) has been considered the gold standard technique for pain management after thoracic surgery. Although TEA provides effective pain control in most cases, the invasiveness of the procedure can limit its use in a patient on anticoagulants. There are also other limitations, such as need for skilled care providers to manage thoracic epidural catheter, sympathetic blockade, respiratory depression, and urinary retention [9]. These risks are a significant limiting factor in the utilization of TEA for pain control.

The significant risks associated with TEA have drawn providers to opt for other loco-regional analgesia techniques such as thoracic paravertebral block (TPVB), intercostal nerve block (ICNB), serratus anterior plane block (SAPB), and erector spinae plane block (ESPB). There are many studies on these loco-regional analgesia techniques in the literature; however, there is a lack of clear consensus on the safest and most efficacious technique for various procedures. Currently, the type of loco-regional analgesia a patient receives is often determined by provider preferences and an assessment of the patient’s comorbidities [9].

SAPB has gained popularity amongst providers and surgeons as a desirable form of analgesia in VATS due to positive post-operative patient outcomes such as improved pain control, higher spirometry measurements, and lower incidence of hypotension, to name a few [10]. The aim of this narrative review is to synthesize the current literature on SAPB in VATS to determine clinical safety and efficacy. Furthermore, the authors will explore the risks, benefits, and side effect profile of other types of loco-regional analgesia used in VATS to compare and contrast against SAPB.

## 2. Methods

A comprehensive search was conducted using Cochrane, PubMed, Google Scholar, and Embase databases on 28 January 2025 using the following terms: “video-assisted thoracoscopic surgery”, “loco-regional analgesia”, “serratus anterior plane block”, “pain management following VATS”, and “enhanced recovery after surgery.” The search included date ranges from 2000 to 2025. All studies that were written in the English language, on human subjects, and relevant to SAPB for pain control after VATS were considered for use within this manuscript. Systematic reviews, meta-analyses, and randomized control trials were given preference in the literature search due to their high quality of evidence. Recent studies comparing SAPB to other forms of loco-regional anesthesia were highlighted and outlined in a comparison table.

## 3. Anatomy and Physiology of SAPB

The serratus anterior is a wide, flat muscle located on the side of the chest wall. It extends from the outer surfaces of the first to ninth ribs and attaches to the medial border of the scapula on the same side [11,12]. The serratus anterior is closely associated with several muscles and nerves in the thoracic region. Its upper portion is covered by the pectoralis major and the pectoralis minor, while its inner portion is adjacent to the intercostal muscles [11,13].

The thoracic spinal nerve roots emerge from the intervertebral foramen and divide into two branches: the dorsal branch, which innervates the skin and muscles of the paravertebral region, and the ventral branch, which continues laterally as part of the intercostal nerve. At the mid-axillary line, the intercostal nerve gives rise to the lateral cutaneous branch, which passes through the intercostal and serratus anterior muscles to supply the skin and muscles of the lateral chest wall [11,14]. The long thoracic nerve, running along the surface of the serratus anterior and accompanying the lateral thoracic artery, provides motor innervation to the muscle. Similarly, the dorsal thoracic nerve also courses along the surface of the serratus anterior at the mid-axillary line [11].

**Figure 1:**
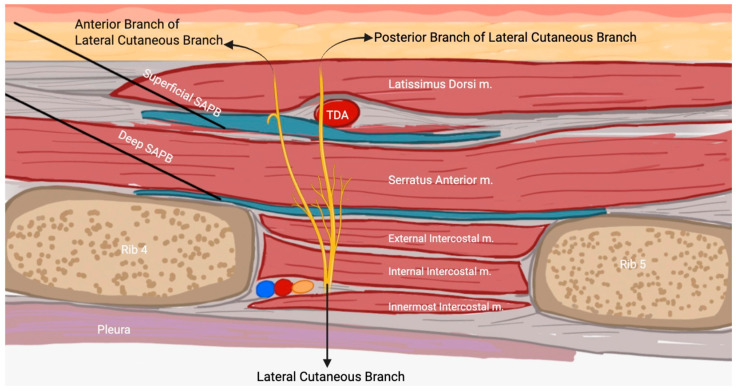
Anatomy for SAPB with needle insertion (black lines). Local anesthetic spread is represented in blue. Superficial SAPB is demonstrated between the latissimus dorsi and serratus anterior muscles. Deep SAPB is underneath the serratus anterior muscle and between the intercostal muscles. Lateral cutaneous nerve branches are shown in yellow and labeled within the figure. TDA = thoracodorsal artery.

The serratus anterior receives arterial supply from the lateral thoracic artery, the superior thoracic artery (supplying the upper portion), and the thoracodorsal artery. The SAPB is capable of blocking the lateral cutaneous branch of the intercostal nerve along with the long thoracic and thoracodorsal nerves. This extensive coverage likely explains its effectiveness in anesthetizing the anterolateral chest wall, which is often difficult to achieve with other regional anesthesia techniques, such as TEA or TPVB [11]. Landmark anatomy in SAPB is highlighted in Figure 1.

### 3.1. SAPB Procedure

The SAPB effectively manages pain from lateral and anterolateral rib fractures, thoracic surgeries, and breast procedures, including post-mastectomy pain [14,15,16,17,18]. It is particularly beneficial for trauma patients with multiple rib fractures requiring opioid analgesia to support respiratory function. SAPB is absolutely contraindicated in patients with a local anesthetic allergy or an active soft tissue infection at the injection site. Relative contraindications include anatomical factors that hinder ultrasound visualization, such as scarring, fibrosis from prior thoracic surgeries, or subcutaneous air due to trauma. However, given its superficial placement and low risk of vascular injury, therapeutic anticoagulation is typically not a barrier to performing this block in experienced hands [17].

For optimal imaging of superficial structures, a high-frequency linear transducer (e.g., 5–10 MHz or 6–13 MHz) with a larger footprint is recommended, ensuring clear needle visualization throughout the procedure. Since SAPB requires a relatively large volume of anesthetic, patients should have an IV line in place and be on cardiac monitoring to detect potential local anesthetic systemic toxicity (LAST). Additionally, lipid emulsion therapy should be readily available in case of toxicity management [17]. Current research indicates that lidocaine, ropivacaine, and bupivacaine are the most commonly used local anesthetics for SAPB. Among these pharmacological agents, ropivacaine and bupivacaine are preferred due to their high safety profiles [11].

**Figure 2:**
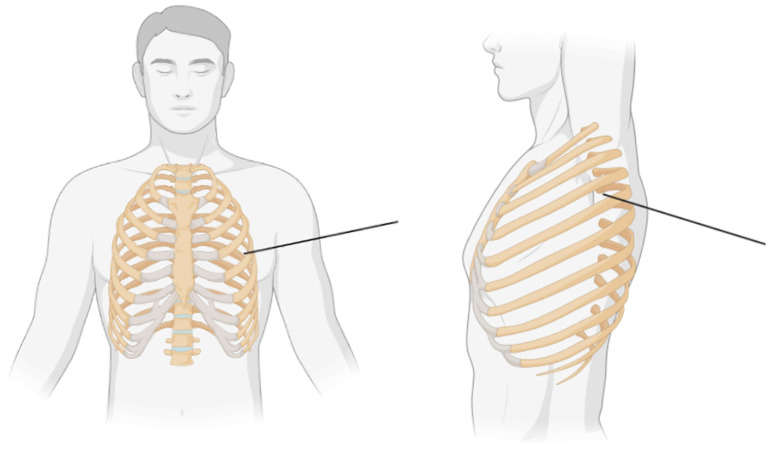
Simple anatomic figure demonstrating the injection site of local anesthetic at the fifth rib in the mid-axillary line (black line) in the anterior view (**left image**) and lateral view (**right image**). Figure created using BioRender, Macie A. (2025).

Different SAPB approaches—superficial, deep, modified, and continuous—offer tailored pain relief depending on the procedure and patient needs, each with its own advantages for effective analgesia. The superficial SAPB involves placing the patient in a supine position, using ultrasound to locate the fifth rib at the mid-axillary line, and injecting local anesthetic between the latissimus dorsi and serratus anterior muscles. This provides analgesia from T2-T9, effectively covering the anterolateral chest wall [11,15]. A simple anatomic image of the location of the injected anesthetic for superficial SAPB is shown in Figure 2.

The deep SAPB targets the space between the serratus anterior and intercostal muscles. The ultrasound probe is placed under the outer clavicle and moved to the fifth rib before injecting local anesthetic. While the superficial approach provides broader coverage, the deep block may be more effective for specific pain patterns [11,19]. The modified SAPB positions the ultrasound probe obliquely from the second to the sixth rib at the posterior axillary line. This approach, ideal for breast reconstruction with a latissimus dorsi flap, blocks the thoracodorsal nerve and pectoral plexus, which standard SAPB techniques do not reach [11,20]. For continuous SAPB, the patient is placed laterally for sterility. A catheter is inserted at the fourth or sixth rib, allowing for continuous local anesthetic infusion. This method is particularly effective for prolonged pain control after VATS, thoracotomy, or rib fractures, reducing the needs for opioids [11,21,22,23,24,25,26].

### 3.2. SAPB Compared to TEA and TPVB

Compared to TEA and TPVB, SAPB offers a simpler, safer alternative with fewer complications while still providing effective analgesia, making it a valuable option for thoracic surgery patients. TEA has been the standard for post-thoracic surgery pain control but is technically complex, requiring catheter placement, continuous infusion, and careful monitoring. It also carries risks like dural puncture, hypotension, and LA toxicity, with contraindications such as spinal surgery history and anticoagulation. SAPB offers a simpler, safer alternative with fewer complications, providing effective analgesia, better hemodynamic stability, and reduced opioid use, making it a valuable option for thoracic surgery patients [11,16,24,27,28]. TPVB provides effective analgesia by injecting local anesthetic into the paravertebral space, preserving contralateral respiratory and sympathetic function. While TPVB has similar complications to TEA, spinal cord-related issues are more common with TEA, where pulmonary complications are more frequent with TPVB. SAPB and TPVB offer comparable pain relief for VATS, but SAPB is simpler and avoids multiple injections, which are often needed for adequate TPVB coverage [10,11,29,30,31].

## 4. Video-Assisted Thoracic Surgery (VATS)

VATS is a minimally invasive surgical procedure used for the diagnostic and therapeutic management of numerous intrathoracic conditions that require surgery. Since its inception as an alternative to traditional open thoracotomy decades ago, VATS has continued to evolve and rise in popularity. This is largely due to the improved postoperative outcomes of VATS when compared to open thoracotomy, including reduced postoperative pain, shorter hospital stays, and reductions in several postoperative complications [32]. However, some surgeons, like Gulati et al., argue that the choice between VATS and open thoracotomy for lobectomy may not be quite as clear as others indicate, citing the need to prioritize patient safety and survival [32]. Ultimately, it comes down to the operating physician’s decision-making on a case-by-case basis.

VATS has a litany of diagnostic and therapeutic indications including various intrathoracic biopsies, cancer staging, pulmonary resection, esophagectomy, chest trauma, pericardial drainage, etc. [33]. The contraindications specific to VATS are surgeon-dependent and situation-specific, relying on the surgeon’s expertise and level of comfort with the procedure. Large, centrally located tumors, challenging anatomy, and inability to tolerate single-lung ventilation are generally agreed to be relative contraindications to VATS. However, inability to tolerate single-lung ventilation can also be a contraindication to any intrathoracic procedure involving the lungs. Other relative contraindications to VATS include severe pleural adhesions, hemodynamic instability, prior talc pleurodesis, previous thoracotomies, intraluminal airway mass, prior thoracic radiation, severe hypoxia, severe COPD, severe pulmonary hypertension, and coagulopathy. VATS is associated with potential complications that are common to any intrathoracic procedure. These complications include postoperative air leak, postoperative pain, hypoxemia, atelectasis, bleeding, infection, and postoperative re-expansion pulmonary edema [32]. Significant bleeding in VATS pulmonary resection is considered a critical complication that requires emergent conversion to open thoracotomy [33]. However, patients who undergo conversion of VATS to open thoracotomy have similar morbidity and mortality rates to patients who undergo open thoracotomy as the primary approach [34].

The VATS procedure technique most often places the patient in the lateral decubitus position, with one-lung ventilation under anesthesia [33]. Adequate oxygenation in one-lung ventilation is often achieved by utilizing double-lumen endotracheal tubes or bronchial blocking [33]. One to four incisions are then made [33,35]. The camera port is usually placed near the mid or anterior axillary line in the seventh or eighth intercostal space. In upper lobectomy, a utility incision is performed at the anterior axillary line near the fifth intercostal space. This is lowered to the sixth or seventh intercostal space in the middle and lower lobectomies. The third and fourth incisions are placed high in the mid-axillary line or low in the chest at the posterior axillary line. Following incision, careful dissection into the thoracic cavity is performed to avoid instrumentation injury to adjacent ribs and intercostal bundles. The camera is inserted into the port, and the respective procedure is performed [35]. Alternatively, the uniport approach requires only one incision for visualization, instrumentation, and removal of thoracic contents [36].

Traditionally, VATS postoperative pain management uses IV paracetamol and NSAIDs with the option for opioid analgesia [9]. The emergence of loco-regional analgesia offers another method with increased duration in patients who have undergone VATS. Some of the current loco-regional options for analgesia include TEA, TPVB, ICNB, SAPB, and ESPB [9]. Inadequate perioperative pain control in thoracic surgeries may result in a number of complications like hypoxia, atelectasis, pneumonias, and chronic post-thoracotomy pain [37]. As VATS continues to evolve, it is vital for post-operative analgesic methods to continue evolving to offer patients the best possible outcomes.

## 5. Clinical Studies Evaluating SAPB for VATS

The SAPB has emerged as an effective regional anesthesia technique for providing postoperative analgesia in patients undergoing VATS. The recent literature indicates its numerous advantages, including superior pain control, reduced opioid consumption, a lower incidence of postoperative nausea and vomiting (PONV), and improved pulmonary recovery.

It should be noted that there are clear benefits when comparing the efficacy of SAPB to other established regional anesthesia techniques for VATS, such as the TEA and the TPBV. In this regard, SAPB provides comparable, if not superior, analgesia to traditional methods for postoperative pain control. A meta-analysis by Li et al. demonstrated a significant reduction in postoperative pain following VATS in patients who received SAPB compared to those who underwent control methods (no block, placebo, or local infiltration anesthesia) [38]. These findings were solidified by Park et al., whose randomized controlled trial showed that patients receiving pre-operative SAPB experienced significantly lower pain scores than those given a placebo [18]. Similarly, Wang et al. reported in their meta-analysis that SAPB led to markedly lower pain scores 24 h post-surgery [39]. Hanley et al. further expanded on these results, comparing pain scores at rest, with movement, and during coughing at 24 h post-procedure [10]. They found that SAPB recipients consistently reported lower pain scores than those who received TPVB. Additionally, their study noted the presence of a detectable sensory blockade in patients receiving SAPB, a finding lacking in the TPVB group. These findings showcase SAPB as an effective regional analgesic technique for postoperative pain control following VATS.

Regarding postoperative opioid consumption and its associated risk of PONV, research on the effects of SAPB in VATS patients presents mixed findings. Compared to control methods, studies show that patients receiving SAPB prior to VATS require significantly fewer opioids postoperatively for effective analgesia and report a reduced incidence of PONV [18,38,39]. However, when compared to other regional anesthesia techniques, the results become less clear. Both Hanley et al. and Ülger et al. found no significant difference in opioid or additional analgesic requirements between patients receiving SAPB and those receiving TPVB prior to VATS [10,40]. Similarly, Lusianawati et al. reached similar conclusions when comparing opioid usage and associated PONV in patients receiving SAPB versus TEA [41]. In contrast, Xiang et al. found that patients who received TEA or TPVB required significantly fewer opioids than those who did not receive a regional anesthetic [42]. However, they observed no significant difference in opioid consumption between patients who received SAPB versus those who did not receive a regional block. Additionally, Zhang et al. reported that patients who received TPVB had significantly lower opioid consumption than those who received either deep or superficial SAPB [43]. However, Zhang also observed that patients with superficial SAPB pressed their patient-controlled intravenous anesthesia (PCIA) devices fewer times and required smaller doses than those receiving TPVB during the first 12 h after VATS. While SAPB demonstrates a clear reduction in opioid consumption and associated PONV when compared to controls, its relative effectiveness compared to other regional anesthesia techniques remains inconclusive, with varying results across studies.

Regarding patient rehabilitation after thoracic surgery, several studies have evaluated the effects of SAPB on pulmonary and functional recovery. Gao et al. found that patients who received SAPB post-VATS demonstrated significantly higher spirometry measurements compared to those who received only PCIA [44]. Additionally, SAPB patients experienced fewer postoperative pulmonary complications, including hypoxemia, pneumonia, and atelectasis. They also reported greater comfort during breathing exercises. Hanley et al. further highlighted SAPB’s benefits, showing that patients who received SAPB had a greater walking distance on the first postoperative day compared to those who received TPVB [10]. These findings suggest that effective pain management with SAPB can significantly enhance both pulmonary and functional recovery after VATS. However, no significant differences were observed in the length of hospital stay between patients receiving SAPB, TPVB, or TEA [18,38,39,45]. While SAPB may improve recovery, its impact on hospitalization duration may be comparable to other techniques.

SAPB is emerging as a promising alternative to TEA and TPVB for antero-lateral chest wall analgesia due to its more favorable side effect and complication profile. As described by Chen et al., TEA offers excellent analgesia but carries the highest risk of hemodynamic fluctuations, epidural hematoma, local anesthetic toxicity, and urinary retention [11]. It is also contraindicated in patients with abnormal coagulation, either due to underlying disorders or the use of anticoagulant medications. While TPVB has a smaller side effect profile, it is associated with pulmonary complications such as pleural puncture, pneumothorax, and local anesthetic systemic toxicity due to its proximity to large vascular structures. In contrast, SAPB, being a more superficial technique, is not associated with these complications. When comparing these techniques, several studies highlight their respective complication profiles. Lusianawati et al. found that patients receiving SAPB had a significantly lower incidence of postoperative hypotension compared to those receiving TEA [41]. Ülger et al. noted the ease and speed of SAPB application, facilitated by its easy visualization under ultrasound guidance, making it quicker to perform than other regional blocks [40]. This finding is supported by Baytar et al., who observed that SAPB had a shorter application time compared to TPVB [45]. Selected studies and their findings are described in Table 1. In conclusion, the findings detailed above present evidence towards SAPB offering a safer and more efficient option to TEA and TPVB in providing antero-lateral chest wall analgesia for VATS.

## 6. Discussion

VATS, a commonly utilized minimally invasive procedure, involves incisions through multiple layers including the skin, subcutaneous tissue, muscles, and parietal pleura. The deep incisions required are incredibly painful and can hinder timely patient recovery if the patient’s pain is poorly controlled [7,8]. SAPB is a form of loco-regional analgesia that demonstrates adequate pain control, significant reductions in pain, reduced postoperative opioid consumption, and improved pulmonary and functional recovery after VATS [10,18,38,39,44]. It is preferred that SAPB is performed prior to incisions needed for VATS. This allows for optimization of analgesic outcomes. While SAPB following VATS is not contraindicated, the common practice utilizes the analgesic prior to surgery (or prior to extubation from general anesthesia) to decrease pain generated from surgical incisions [7,8].

A key strength of SAPB is the decreased number of side effects reported with its use. This is likely due to the superficial nature of the block that leads to decreased risk of injury to deeper structures with analgesia [40,45]. Additionally, a randomized control trial by Hanley et al. found that patients who received SAPB had better pain controls at rest, movement, and coughing at 24 h compared to TPVB [10]. Throughout the literature reviewed and analyzed in this study, there is no indication that SAPB is inferior to other forms of loco-regional anesthesia for pain control following VATS. Additionally, SAPB has a shorter application time compared to TPVB which can lead to quality improvement measures on efficiency during VATS procedure [40,45]. The ease and duration of administering regional anesthesia is not typically a reported variable in research studies. This could be attributed to different physicians performing the anesthesia procedures within a trial population as well as individual body habitus and other factors not easily accounted for. However, the finding of shorter application time of SAPB by Ülger et al. and Baytar et al. is an interesting and important consideration for future research studies [40,45].

Overall, SAPB has been shown to be safe and effective for post-operative pain control in those undergoing VATS. However, findings are less clear when evaluating for superiority between SAPB, TPVB, and TEA for pain control and postoperative opioid consumption. Other randomized control trials have been unable to show a statistically significant difference in postoperative pain control between SAPB, TEA, and TPVB [40,41,43]. When assessing postoperative opioid consumption, patients who receive any type of regional anesthetic block have a significantly reduced consumption of opioids [41]. Further research is needed on VATS postoperative pain outcomes, opioid consumption, pulmonary function, length of hospital stay, and time of administration to fully delineate whether there is a superior type of loco-regional analgesia indicated for VATS.

## 7. Conclusions

In summary, SAPB is a safe and effective option for pain management following VATS. Multiple randomized control trials and a meta-analysis have evaluated the efficacy and safety of SAPB in comparison to control anesthetics or other loco-regional techniques. No significant difference has been identified in length of hospital stay and postoperative opioid consumption amongst SAPB and other loco-regional analgesia techniques such as TEA and TPVB. However, the SAPB has been cited to be an easier and safer technique to perform with a shorter application time. This narrative review provides up-to-date evidence supporting the use of SAPB for pain control in VATS due to its efficacy, safety, and relative ease of performance. These findings are promising for the field of minimally invasive surgery and enhanced recovery after surgery pathways.

## Figures and Tables

**Table 1:** Comparative Studies of SAPB, TEA, and TPVB.

Author (Year)	Study Design	Findings	Conclusions
Hanley et al. (2020) [10]	Single-center, double-blind randomized controlled non-inferiority trial comparing ultrasound-guided continuous deep SAPB with surgically placed continuous TPVB in patients undergoing VATS. Evaluated whether SAPB is non-inferior to TPVB in reducing forty-eight-hour postoperative opioid consumption, improving pain scores and functional recovery metrics.	SAPB demonstrated non-inferiority to TPVB in postoperative opioid consumption. SAPB demonstrated significantly better pain scores at rest, movement, and coughing at twenty-four hours compared to TPVB. SAPB improved day one postoperative walking distance, with no differences noted in side effects or hospital stay length.	SAPB is an effective, opioid-sparing alternative to TPVB for perioperative analgesia in VATS, offering comparable pain control with enhanced functional recovery.
Ülger et al. (2024) [40]	Prospective, randomized, double-blind equivalence study compared the efficacy of combined deep and superficial SAPB (C-SAPB) with TPVB for postoperative pain management in sixty patients undergoing VATS. Variables tested were pain scores, opioid consumption, side effects, and patient satisfaction over forty-eight hours postoperatively.	Both C-SAPB and TPVB demonstrated similar analgesic efficacy, with no significant differences in pain scores, opioid consumption, or patient satisfaction. C-SAPB had a slightly shorter application time.	C-SAPB is an effective and safe alternative to TPVB for postoperative pain management in VATS, offering comparable pain relief with a potentially simpler and faster application process.
Lusianawati et al. (2023) [41]	Systematic review and meta-analysis including six randomized controlled trials comparing SAPB and TEA for thoracic and breast surgeries. Aimed to compare the efficacy and safety of SAPB and TEA, evaluating postoperative pain, incidence of hypotension, and PONV.	No significant difference in visual analogue scale pain scores between SAPB and TEA at various postoperative times. SAPB had a significantly lower incidence of hypotension compared to TEA. No significant difference in PONV incidence between the two techniques.	SAPB is a safe and equally effective alternative to TEA for pain management in thoracic and breast surgeries, with a lower incidence of hypotension. Larger studies are needed to confirm these findings.
Xiang et al. (2024) [42]	Single-center, retrospective cohort study including 2159 patients undergoing VATS, divided into four groups based on regional block type: GA, TEA, TPVB, and SAPB, with matching for age, sex, ASA physical status, and surgery duration. Evaluated intraoperative opioid-sparing effects of TEA, TPVB, and SAPB compared to general anesthesia alone in VATS patients.	TEA and TPVB significantly reduced intraoperative opioid consumption. SAPB did not show significant opioid-sparing effects. No significant differences in time-to-first rescue analgesic or postoperative complications between groups.	TEA and TPVB significantly reduced intraoperative opioid consumption, making them part of an optimal analgesic strategy for VATS. SAPB did not show significant opioid-sparing effects. Further studies are needed to explore opioid-free anesthesia options for VATS.
Zhang et al. (2022) [43]	Randomized controlled trial including seventy-four patients undergoing VATS, divided into three groups: deep serratus plane block (DSPB), superficial serratus anterior plane block (SSPB), and paravertebral nerve block (TPVB). Compared analgesic efficacy of DSPB, SSPB, and TPVB for postoperative pain management in VATS via opioid consumption, pain scores, and patient satisfaction.	No significant postoperative pain scores among the groups. TPVB reduced intraoperative opioid consumption, while SSPB showed lower opioid requests and dosing during the first twelve hours postoperatively. DSPB and SSPB were easier to perform.	All three techniques provided effective postoperative analgesia, with PVB offering better intraoperative pain control. DSPB and SSPB are easier to perform and could be viable alternatives to PVB, but further research on continuous infusion techniques is needed.
Baytar et al. (2021) [45]	A prospective, randomized, double-blind study compared ultrasound-guided SAPB and TPVB in VATS patients. Determined whether SAPB is as effective as TPVB for pain control in VATS. Secondary objectives included assessing satisfaction, block application time, analgesic requirement, postoperative complications, and hospital stay length.	No significant differences in pain scores between groups at various time points. TPVB group had lower tramadol consumption. SAPB had significantly shorter block application time. No significant differences in first analgesic requirement, hospital stay, satisfaction, or complications.	SAPB is not inferior to TPVB for postoperative pain control in VATS, offering a faster block application time and serving as a viable alternative to TPVB.
